# Detection of antibodies directed at *M. hyorhinis *p37 in the serum of men with newly diagnosed prostate cancer

**DOI:** 10.1186/1471-2407-11-233

**Published:** 2011-06-10

**Authors:** Cydney Urbanek, Steve Goodison, Myron Chang, Stacy Porvasnik, Noburo Sakamoto, Chen-zhong Li, Susan K Boehlein, Charles J Rosser

**Affiliations:** 1 Department of Urology, The University of Florida, Gainesville, Florida, 32610, USA; 2 Department of Surgery, The University of Florida, Jacksonville, Florida, 32209, USA; 3 Department of Biostatistics, The University of Florida, Gainesville, Florida, 32610, USA; 4 Department of Biomedical Engineering, Florida International University, Miami, Florida, 33174, USA; 5 Department of Biochemistry, The University of Florida, Gainesville, Florida, 32610, USA

**Keywords:** *Mycoplasma hyorhinis*, ELISA, cancer, prostate

## Abstract

**Background:**

Recent epidemiologic, genetic, and molecular studies suggest infection and inflammation initiate certain cancers, including cancers of the prostate. Over the past several years, our group has been studying how mycoplasmas could possibly initiate and propagate cancers of the prostate. Specifically, *Mycoplasma hyorhinis *encoded protein p37 was found to promote invasion of prostate cancer cells and cause changes in growth, morphology and gene expression of these cells to a more aggressive phenotype. Moreover, we found that chronic exposure of benign human prostate cells to *M. hyorhinis *resulted in significant phenotypic and karyotypic changes that ultimately resulted in the malignant transformation of the benign cells. In this study, we set out to investigate another potential link between mycoplasma and human prostate cancer.

**Methods:**

We report the incidence of men with prostate cancer and benign prostatic hyperplasia (BPH) being seropositive for *M. hyorhinis*. Antibodies to *M. hyorhinis *were surveyed by a novel indirect enzyme-linked immunosorbent assay (ELISA) in serum samples collected from men presenting to an outpatient Urology clinic for BPH (N = 105) or prostate cancer (N = 114) from 2006-2009.

**Results:**

A seropositive rate of 36% in men with BPH and 52% in men with prostate cancer was reported, thus leading us to speculate a possible connection between *M. hyorhinis *exposure with prostate cancer.

**Conclusions:**

These results further support a potential exacerbating role for mycoplasma in the development of prostate cancer.

## Background

Recent studies suggest infection and inflammation initiate certain cancers including cancers of the prostate [1-5]. According to the American Cancer Society, approximately 20% of all worldwide cancers are caused by infections [6]. These infectious agents may directly induce tumorigenesis through viral or bacterial protein products that have oncogenic effects or indirectly through a local chronic and progressive inflammatory response [7-9]. There is a paucity of information regarding the role of mucosal bacteria in contributing to malignancies of the prostate. One class of bacteria that is of particular interest is the *Mollicutes*. Mycoplasmas (class *Mollicutes*) are tiny, pleomorphic, wall-free, prokaryotic organisms that can reside either on the eukaryotic cell membrane or inside the cell. They are the smallest organisms (0.2-0.3 μm) capable of self-replication [10] with genomes of approximately 580-1200 kBp. Several mycoplasmas have been well documented as human pathogens [11,12], however, it is conceivable that many mycoplasmal infections may go unidentified since numerous species can grow for extended periods of time in close interaction with mammalian cells without producing obvious cytopathic effects or noticeable symptoms.

A modern understanding of the latency of cancer and the emerging role of microbes in carcinogenesis raises the question of whether mycoplasmas can induce malignant transformation and thus warrants further investigation [13,14]. Studies of leukemic patients in the mid-1960s raised the possibility of an association between mycoplasma infection and the development of leukemia [13]. Over the past several years much work has been devoted to identifying a mechanism by which mycoplasmas can transform cells. Specifically, our group reported that infection of benign human prostate cells, BPH-1, for 19 weeks resulted in anchorage-independent growth, increased migration and invasion, accumulation of chromosomal aberrations and polysomy, and the ability to form xenograft tumors in athymic mice. This was the first report describing the capacity of *M. hyorhinis *infection to cause the malignant transformation of benign human epithelial cells [15]. Furthermore, our group demonstrated that cells subjected to a single *M. hyorhinis *protein, p37, resulted in increased proliferation, significant genomic changes, and an enhanced invasive capability [16,17].

Working independently, several groups have detected the *M. hyorhinis *p37 protein in cancer patients. The p37 protein was first described in an effort to identify human cell antigens that elicit tumor-specific antibodies. Fareed et al. [18] analyzed the immune response in a group of cancer patients who were immunized intralymphatically with tumor cell extracts. Sera samples from patients who were in a state of tumor regression showed measurable antibody titers against several antigens, including a 38-kDa protein. These antigens were not detected in those patients whose tumors failed to regress. The 38 kDa antigen was later identified as a mycoplasmal protein from *M. hyorhinis*. The protein was designated as p37 [19,20]. In this study, we developed an indirect ELISA to investigate the presence of serum antibodies (IgG and IgM) against *M. hyorhinis *p37 in men with newly diagnosed localized prostate cancer.

## Methods

### Patient Serum Specimens

After Institutional Review Board approval and signed informed consent, serum samples were prospectively collected from 321 men presenting to the Department of Urology University of Florida for evaluation of BPH or prostate cancer from 2006-2009. Briefly, 5-ml of whole blood was collected in a serum separating tube from each subject. Within 30 minutes, the tube was placed in the centrifuge and spun for 15 minutes at 2400 rpm as dictated in our standard operating procedures of our departmental tissue bank. Fifty microliter of serum was pipetted into multiple 1-ml cryogenic vials, snap-frozen and stored at -80°C for future use. Hospital records were reviewed for demographic, clinical and pathologic data. A total of 219 subjects (N = 114, BPH and N = 105, prostate cancer) with adequate hospital records and banked serum samples comprised the study cohort.

### Expression and purification of recombinant p37

**Figure 1:**
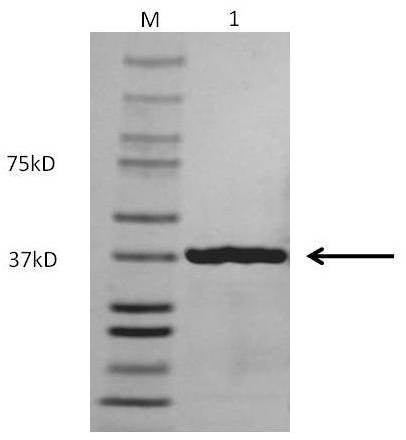
**Purification of recombinant *M. hyorhinis *p37 protein by affinity chromotography**. *M. hyorhinis *p37 (MH38-113) was expressed in *E. coli *and purified as described previously [16]. Sonicated cell lysate of *E. coli *was applied to a cobalt affinity column, and the bound protein was eluted with 150 mM imidazole. A total of 25 μg eluate was electrophoresed in a 12% SDS-PAGE gel, and stained with Coomassie blue. M, Prestained BenchMark Protein ladder (kDa); 1, purified recombinant protein. The arrow indicates the position of the recombinant protein.

*M. hyorhinis *p37 (MH38-113) was expressed and purified as previously described [16]. Briefly, plasmids were transformed into BL21(DE3)pLysS E. coli cells. The transformation was used to inoculate 1-L LB media with 100 mg L21 ampicillin and cultured at 37°C until the OD 600 nm was 0.7-1.0. Cells were induced using 1 mL of 1M isopropyl b-D-1 thiogalactopyranoside and allowed to express for three hours. Cells were lysed by vortexing the pellet in 1/10 the original volume of 20 mmol/L phosphate buffer (pH 7.8) followed by a sonication for three 15-second cycles. The resulting crude cell lysate was centrifuged at 40,000 × g for 20 minutes at 4°C to remove cell debris. The clear supernatant (soluble cellular extract) was subjected to ion exchange chromatography using the Econo System (Bio-Rad). A 5 mL Bio-Rad Econo-Pac S cation exchange column was attached to the bottom of a 50 mL Bio-Rad anion exchange Q column, and equilibrated with 20 mmol/L sodium phosphate buffer (pH 7.95) at a flow rate of 2.5 mL/min. Approximately 125 mg of soluble cellular extract were loaded on the column. The flow-through containing *M. hyorhinis *p37 was adjusted to pH 6.1 with 2 mol/L acetic acid, and loaded on a 5 mL cation exchanger, Bio-Rad Econo-Pac S cartridge, equilibrated with 20 mmol/L sodium acetate, pH 6.1 (buffer A). The column was washed with 5% buffer B [20 mmol/L sodium acetate (pH 6.1), 1 mol/L NaCl] and the *M. hyorhinis *p37 protein was eluted with 15% buffer B. The eluted sample was then concentrated using a Centriprep 10 spin column (Millipore, Bedford MA). Purity was confirmed by 10% SDS-PAGE stained with Coomassie Blue (Figure 1). Concentrations were calculated by absorbance at 280 nm using a calculated extinction coefficient of 54,620 M^-1^cm^-1^.

### Indirect ELISA

A 96-well plate was coated with 100 μl of diluted *M. hyorhinis *p37 (0.5 μg/well). Plates were incubated for at least one hour at room temperature. The plate was washed four times with TBS/Tween 20 (50 mM Tris-HCl, pH 7.5, 150 mM NaCl, 5 mM MgCl2, 0.5 mM CaCl2, 0.05% (v/v) Tween 20. Next, the plates were blocked with 300 μl of TBS/1% BSA and incubated for 1 hour. Again the plates were washed as described above. Based on our preliminary studies (data not shown), thawed human sera samples were diluted 1/100 with TBS and 100 μl of each diluted human sera were then added to the 96-well plate in duplicate. Plates were incubated for another one hour at room temperature. Plates were washed four times as described previously followed by the addition of 100 μl of diluted anti-human antibody (1/100) conjugated to alkaline phosphatase. Plates were incubated for one hour at room temperature then washed again. Next, the plate was washed once with TBS containing no Tween 20, and 100 μl of freshly made p-NPP in development buffer was added.

We used a naturally occurring antibody as an internal control. The disaccharide, Gal1α1,3 Gal, is present in all humans, and IgG antibodies to Gal1α1,3 Gal are found to be present in high titers in the serum of every normal individual, and are constantly produced throughout life [21]. Gal1α1,3 Gal was purchased from Sigma Chemical Co., St. Louis, Missouri, USA. Test serum samples were also assayed with Galα1,3 Gal as the substrate (positive control) coupled to bovine serum albumin (BSA). BSA alone served as the negative control. Plates were read at 405 nm in a plate reader. All of the sera tested showed a strong positive response against Galα1,3 Gal-BSA and no response to BSA alone (data not shown).

### Statistical analysis

We used the Wilcoxon rank-sum test to compare the O.D. values and PSA values in the prostate cancer group to those in the BPH group. Since we are testing the hypothesis that the O.D. values and PSA values in the prostate cancer group are higher than that in the BPH group, all reported *p*-values are one-sided. The one-sided Wilcoxon rank-sum test is also used to assess the correlation between O.D. values and clinical parameters. We defined a diagnostic test (positive indirect ELISA assay vs. negative indirect ELISA assay) using a cutoff value of O.D. selected to maximize the sum of the sensitivity and specificity of the test [22]. All data analyses were performed using SAS software version 9.1.3.

## Results

Sera from a total of 219 subjects (N = 114, BPH and N = 105, prostate cancer) comprised our study cohort. The cohorts' demographic, clinical and pathologic features are summarized in Table 1. The two study groups (BPH and prostate cancer) were well matched for age and race. Serum PSAs were higher in the cancer group versus the BPH group (5.7 +5.1 vs. 0.9+1.8, *p *< 0.0001). Of the 105 subjects with prostate cancer, the majority of subjects presented with low risk prostate cancer; clinical T1c prostate cancer (69%), serum PSA <10 ng/ml (86%) and Gleason score 3+3 = 6 (65%). A small percentage of these subjects (n = 38) underwent a radical prostatectomy for definitive therapy. Table 2 describes clinicopathologic features of the prostatectomy cohort. Median follow-up of our entire cohort was 18.1 months. In this short follow-up, 5% of the patients experienced biochemical recurrence.

**Table 1:** Demographic and clinicopathologic characteristics of study cohort

	BPH	Cancer
	N = 114	N = 105
Median Age (range, y)	60 (30-86)	60 (41-79)
Race		
White	73 (64)	72 (69)
African American	30 (26)	17 (16)
Other	11 (10)	16 (15)
Median Serum PSA (ng/ml)	0.9+1.8	5.7 ± 5.1
Clinical Stage		
T1c	n/a	74 (70%)
T2a	n/a	26 (25%)
T2b	n/a	1 (1%)
T2c	n/a	2 (2%)
T3a/b	n/a	1 (1%)
T2aNxM1	n/a	1 (1%)
Gleason Score		
6	n/a	68 (65%)
7	n/a	23 (22%)
>7	n/a	14 (13%)

**Table 2:** Demographic and clinicopathologic characteristics of 38 study subjects who underwent radical prostatectomy

	Cancer
	N = 38
Median Age (range, y)	63 (47-72)
Race	
White	24 (63)
African American	12 (32)
Other	2 (5)
Median Serum PSA (ng/ml)	9.2 ± 4.7
Pathologic Stage	
pT2	31 (82%)
pT3a	3 (8%)
pT3b	4 (10%)
N0	36 (95%)
N1	2 (5%)
Gleason Score	
6	19 (50%)
7	13 (34%)
>7	6 (16%)

**Figure 2:**
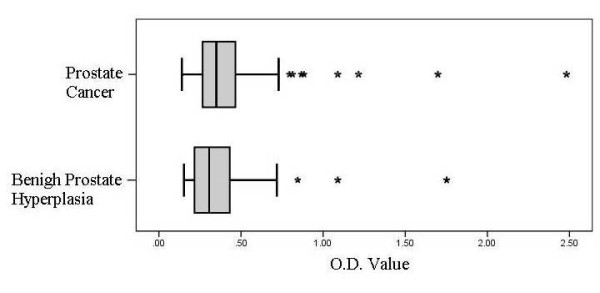
**Box plots of O.D. value detecting *M hyorhinis *serum antibodies**. The distributions of O. D. values are presented in a box plot.

**Figure 3:**
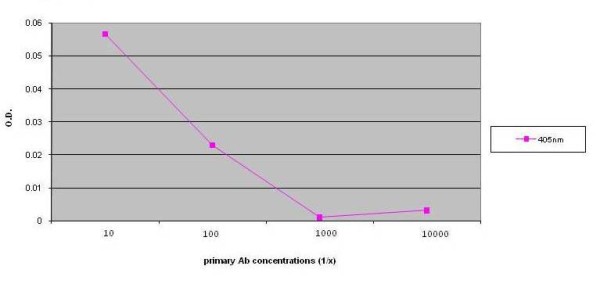
**Detection limits of novel indirect ELISA for *M. hyorhinis *serum antibodies**. Varying concentrations of *M. hyorhinis *antibodies were added to our indirect ELISA to illustrate the range of detection.

Indirect ELISA assays were performed on all 219 sera samples in duplicate. The median O.D. value for the BPH group was 0.31 whereas the median O.D. value was 0.35 in the prostate cancer group (*p *= 0.035). The distributions of O. D. values are presented in a box plot (Figure 2). Through further data analysis we determined an optimum O.D. cut off value to distinguish a positive indirect ELISA assay (i.e., harboring antibodies to *M. hyorhinis *p37) of > 0.348. Utilizing this O.D. cut off value, 41 out of the 114 (36%) BPH subjects and 55 of 105 (52%) prostate cancer subjects had antibodies to *M. hyorhinis *p37 (*p *= 0.014) (Table 3). Figure 3 depicts the sensitivity of this novel indirect ELISA assay towards *M. hyorhinis *p37 antibodies. The O.D. values were not significantly associated with clinical stage of prostate cancer patients (*p *= 0.39). The O.D. values of prostate cancer patients with Gleason score 7 or higher were significantly higher than that with Gleason score 6 (*p *= 0.016). Biochemical recurrence was not associated with a positive ELISA assay (*p *> 0.05).

**Table 3:** Summary of serum Ig *M.hyorhinis *p37 antibody detection by Indirect ELISA

	-Ig Mh	+ Ig Mh	*p*-value
Benign Patients (N = 114)	73/114 (64%)	41/114 (36%)	0.014
Cancer Patients (N = 105)	50/105 (48%)	55/105 (52%)	

## Discussion

In this study, we provide evidence supporting a potential role for mycoplasma in the initiation and/or propagation of human cancers. Fifty-two percent of men with prostate cancer harbored antibodies to *M. hyorhinis *while only thirty-six percent of men with the benign prostate condition, BPH, were found to have antibodies to *M. hyorhinis (p *= 0.014). If antibodies to *M. hyorhinis *are present, then we assume that these individuals were exposed to *M. hyorhinis *within their life time. This is not unexpected since *M. hyorhinis *is a ubiquitous organism. Other intriguing links between cancer and *M. hyorhinis *exposure have been recently elucidated. A group from Japan reported that 48% of tumors from patients with gastric cancer were positive for *M. hyorhinis *[23]. In addition, a study from China strongly supports a link between *M. hyorhinis*, p37 expression and cancer. A monoclonal antibody that specifically recognizes p37 was used to test for reactivity in over 500 paraffin-embedded normal and diseased tissues. The results indicated that 40-53% of gastric, esophageal, and colon carcinoma samples were positive for reactivity with the *M. hyorhinis *p37 monoclonal antibody [24].

Our laboratory has preliminary evidence linking *M. hyorhinis *protein p37 to cancer initiation and/or progression [16,17]. Specifically we demonstrated that recombinant p37 enhanced the invasiveness of two prostate carcinoma and two melanoma cell lines in a dose-dependent manner *in vitro*, but did not have a significant effect on tumor cell growth. These findings could be completely blocked with a neutralizing antibody to *M. hyorhinis *p37 [16]. In a separate study, recombinant *M. hyorhinis *p37 induced a more malignant phenotype in prostate cancer cells PC-3 and DU145 as demonstrated by significant nuclear enlargement, anaplasia, and increased migratory activity. Furthermore, these cells showed differential expression of genes involved in cell cycle, signal transduction and metabolism [17]. Taken together, these studies support a strong association between *M. hyorhinis *p37 epitope expression and cancer that is complex, probably requiring a long latency period, and may be dependent upon specific host factors.

Mycoplasmas are notorious for producing infections that can persist for up to a year or longer [25]. The effects of long-term exposure of mycoplasma on gene expression in mammalian cells have been carefully studied [26]. Gene expression changes were examined following infection of human cervical and prostatic epithelial cells *in vitro *with a panel of mycoplasmas. The changes in expression of 38 key cytokine genes were examined over a period of time ranging from 12 hours to 36 weeks. The results indicated that, even in the absence of apparent changes in cell growth or cell morphology, mycoplasmal infections rapidly altered the expression of many key genes, thus altering numerous important biological functions within cells [26].

Over the past several years much work has been devoted to identifying a mechanism by which mycoplasmas can transform cells. The oncogenic potential of human mycoplasmas, *M. fermentans *and *M. penetrans*, were studied using cultured C3H mouse embryo cells [27]. Transformation with mycoplasma was a multistage process, with distinct phases in promotion and progression towards malignancy. During initial mycoplasmal infection, the effects were reversible (i.e., removal of the mycoplasma restored normal cellular function). However, after chronic infection, the transformation became irreversible. Thus, mycoplasma-mediated oncogenesis had a long latency period and required a chronic persistent infection, as opposed to the acute transformation induced by most oncogenic viruses [26]. Because of this long latency, it is extremely difficult to establish a link between mycoplasmas and cancer through an epidemiologic approach.

Our group has studied the oncogenic potential of *M. hyorhinis *using cultured BPH-1, benign human prostate cells. The immortalized BPH-1 cell line was derived from primary cultures of benign prostatic epithelial cells by introducing SV40T antigen [28] which inactivates both p53 and Rb tumor suppressor genes. Thus we hypothesized that further insult or stress (e.g., chronic mycoplasmal exposure) in these benign prostate cells may render the benign cells susceptible to further genetic damage and to progression along a pathway to malignancy. After being exposed to *M. hyorhinis *for 19 weeks, BPH-1 cells achieved anchorage-independent growth, increased migration and invasion, accumulation of chromosomal aberrations and polysomy and formed xenograft tumors in athymic mice. Transformation with mycoplasma was a multistage process, with distinct phases in promotion and progression towards malignancy [15]. This novel cell transformation model was critical in elucidating the potential of chronic mycoplasmal exposure leading to the development of prostate cancer. Though intriguing, further work is needed to confirm and further explain the role of *M. hyorhinis *in the development and propagation of human prostate cancer.

We report the development of the first indirect ELISA assay for the detection of circulating *M. hyorhinis *antibodies in human serum samples. Overall, *M. hyorhinis *antibody was detected in 44% of our cohort (36% in BPH and 52% in prostate cancer). The percent of IgG and IgM antibodies within the entire pool of antibodies were not determined, neither were antibody titers, however, we did find this system of detecting *M. hyorhinis *antibodies to be reliable and simple, thus allowing further evaluation of this assay in subjects with prostate cancer.

Overall this study provides strong evidence that humans are exposed to *M. hyorhinis *and such exposure may be associated with the development of certain cancers. We recognize that numerous limitations are evident in the current study. First, this is a small, highly selected cohort and thus may not represent the average BPH or prostate cancer patient. Second, confirmation of *M. hyorhinis *within the prostate via immunohistochemical staining or a similar assay was not performed due to limitations of high-quality antibodies directed at *M. hyorhinis*. Third, the association between mycoplasma and prostate cancer is complex and may require a long latency period, a specific set of host attributes, or possibly exposure to a particular strain of mycoplasma, none of these have been clearly identified. Fourth, we do not believe *M. hyorhinis *itself causes malignant transformation, but when present it may further stress cells that have the propensity to become malignant as was evident in our preclinical study [15]. Fifth, our control group was comprised of men with BPH, a benign overgrowth of the prostate. Though it would be ideal to have as a control men without this benign overgrowth of the prostate it is not feasible seeing that the majority of elderly men will have BPH.

We have demonstrated an increased rate of seropositivity to *M. hyorhinis *in men with prostate cancer (52%) compared to men with BPH (36%) presenting to an outpatient Urology clinic, thus providing the first correlation of mycoplasmal exposure and prostate cancer. Though a significant percentage of men with BPH harbored antibodies to *M. hyorhinis *p37, we still believe we have a valid hypothesis. First, *M. hyorhinis *is ubiquitously found in the environment. Second, we believe that prostatic tissue may be exposed to *M. hyorhinis*, which can cause a chronic inflammatory milieu leading to cellular changes. This effect may be instigated by the cell surface protein p37 directly. These cellular changes, when coupled with other cellular stressors, can induce malignant transformation. Thus, it is not surprising to find *M. hyorhinis *in significant proportion of subjects with a benign condition.

## Conclusions

Our current findings coupled with our previous findings of how mycoplasma can transform benign prostatic cells have led us to hypothesize that mycoplasmal exposure may be linked to the initiation and propagation of some prostate cancers. Further epidemiologic studies into this phenomenon are required, but the idea that mycoplasmas can exacerbate, or perhaps even initiate human prostate malignancy may stimulate new thinking on how we prevent, diagnose and treat prostate cancers.

## Abbreviations

kBp: kilobase pair; kDa: kilodalton; ELISA: Enzyme-linked immunosorbent assay; Ig: immunoglobin; BPH: Benign prostatic hyperplasia; OD: optical density; Rb: retinoblastoma.

## Competing interests

The authors declare that they have no competing interests.

## Authors' contributions

All authors have read and approved the final manuscript.

CU, BS processed serum samples and performed ELISA assays; SG, PhD interpreted the data and wrote the manuscript; MC, PhD performed statistical analysis on data

SP, MS performed reported assays; NS, MD processed serum samples

CZL, PhD optimization of ELISA assay; SKB, PhD produced recombinant protein for ELISA assay; Charles JR, MD, MBA designed study, interpreted the data and wrote the manuscript.

## Pre-publication history

The pre-publication history for this paper can be accessed here:

http://www.biomedcentral.com/1471-2407/11/233/prepub

## References

[B1] Radhakrishnan S Lee A Oliver T Chinegwundoh F An infectious cause for prostate cancer BJU Int 2007 99 239 40 10.1111/j.1464-410X.2006.06556.x 17313418

[B2] De Marzo AM Platz EA Sutcliffe S Xu J Gronberg H Drake CG Nakai Y Isaacs WB Nelson WG Inflammation in prostate carcinogenesis Nat Rev Cancer 2007 7 256 69 10.1038/nrc2090 17384581 PMC3552388

[B3] Dennis LK Lynch CF Torner JC Epidemiologic association between prostatitis and prostate cancer Urology 2002 60 78 83 12100928 10.1016/s0090-4295(02)01637-0

[B4] Dennis LK Dawson DV Meta-analysis of measures of sexual activity and prostate cancer Epidemiology 2002 13 72 9 10.1097/00001648-200201000-00012 11805589

[B5] Klein EA Silverman R Inflammation, infection, and prostate cancer Curr Opin Urol 2008 18 3 315 9 10.1097/MOU.0b013e3282f9b3b7 18382242

[B6] Jemal A Siegel R Ward E Murray T Xu J Thun MJ Cancer statistics, 2007. CA Cancer J Clin 2007 57 43 66 10.3322/canjclin.57.1.43 17237035

[B7] Koraitim MM Metwalli NE Atta MA el-Sadr AA Changing age incidence and pathological types of schistosoma-associated bladder carcinoma J Urol 1995 154 1714 6 10.1016/S0022-5347(01)66763-6 7563329

[B8] Hanto DW Frizzera G Gajl-Peczalska KJ Sakamoto K Purtilo DT Balfour HH Jr Simmons RL Najarian JS Epstein-Barr virus induced B cell lymphoma after renal transplantation. Acyclovir therapy and transition from polyclonal to monoclonal B-cell proliferation NEJM 1982 306 913 8 10.1056/NEJM198204153061506 6278307

[B9] Reeves WC Brinton LA Garcia M Brenes MM Herrero R Gaitan E Tenorio F de Britton RC Rawls WE Human papillomavirus infection and cervical cancer in Latin America NEJM 1989 320 1437 41 10.1056/NEJM198906013202201 2541336

[B10] Lo SC Maniloff J, McElheney RN, Finch LR, and Baseman JB Mycoplasmas: Molecular Biology and Pathogenesis 1992 Am. Soc. Microbiol. Press, Washington, DC 525 545

[B11] Paton GR Jacobs JP Perkins FT Chromosome changes in human diploid-cell cultures infected with Mycoplasma Nature 1965 207 992 43 5 10.1038/207043a0 5866523

[B12] Fogh J Fogh H Chromosome changes in PPLO-infected FL human amnion cells Proc Soc Exp Biol Med 1965 119 233 38 14297856 10.3181/00379727-119-30145

[B13] Cimolai N Do mycoplasmas cause human cancerż Can J Microbiol 2001 47 691 697 10.1139/w01-053 11575494

[B14] Feng SH Tsai S Rodriguez J Lo SC Mycoplasmal infectiond prevent apoptosis and induce malignant transformation if interleukin-3-dependent 32D hematopoietic cells Mol Cell Biol 1999 16 12 7995 8002 10.1128/mcb.19.12.7995 PMC84884 10567525

[B15] Namiki K Goodison S Porvasnik S Allan RW Iczkowski KA Urbanek C Reyes L Sakamoto N Rosser CJ Persistent exposure to Mycoplasma induces malignant transformation of human prostate cells PLoS One 2009 4 9 e6872 10.1371/journal.pone.0006872 19721714 PMC2730529

[B16] Ketcham CM Anai S Reutzel R Sheng S Schuster SM Brenes RB Agbandje-McKenna M McKenna R Rosser CJ Boehlein SK P37 Induces Tumor Invasiveness Mol Cancer Ther 2005 4 1031 8 10.1158/1535-7163.MCT-05-0040 16020660

[B17] Goodison S Nakamura K Iczkowski KA Anai S Boehlein SK Rosser CJ Exogenous Mycoplasmal p37 Protein Alters Gene Expression, Growth, and Morphology of Prostate Cancer Cells Cytogenet Genome Res 2007 118 2-4 204 13 10.1159/000108302 18000372

[B18] Fareed GC Mendiaz E Sen A Juillare GJF Weisenburger TH Totanes TJ Novel antigenic markers of human tumor regression Biol Res Mod 1988 7 11 23 3373233

[B19] Ilantzis C Thomson DMP Michelidou A Benchimol S Stanners CP Identification of a Human Cancer Related Organ-Specific Neoantigen Microbiol Immunol 1993 37 119 28 7684807 10.1111/j.1348-0421.1993.tb03188.x

[B20] Dudler R Schmidhauser C Parish RW Wettemhall REH Schmidt T A mycoplasma high-affinity transport system and the in vitro invasiveness of mouse sarcoma cells. EMBO J 1988 7 3971 74 3208756 10.1002/j.1460-2075.1988.tb03283.x PMC454995

[B21] Galili U Rachmilewitz EA Peleg A Flechner I A unique natural human IgG antibody with anti-alpha-galactosyl specificity J Exp Med 1984 160 5 1519 31 10.1084/jem.160.5.1519 6491603 PMC2187506

[B22] Fluss R Faraggi D Reiser B Estimation of the Youden Index and its associated cutoff point Biometrical Journal 2005 47 4 458 472 10.1002/bimj.200410135 16161804

[B23] Sasaki H Igaki H Ishizuka T Kogoma Y Sugimura T Terada M Presence of stretococcus DNA sequence in surgical specimens of gastric cancer Jpn J Cancer Res 1995 86 791 4 7591953 10.1111/j.1349-7006.1995.tb03086.x PMC5920939

[B24] Huang S Li JY Wu J Meng L Shou CC Mycoplasma infections and different human carcinomas World Gastroentero 2001 7 266 269 10.3748/wjg.v7.i2.266 PMC4723534 11819772

[B25] Iverson-Cabral SL Astete SG Cohen CR Totten PA mgpB and mgpC sequence diversity in Mycoplasma genitalium is generated by segmental reciprocal recombination with repetitive chromosomal sequences Mol Microbiol 2007 66 1 55 73 10.1111/j.1365-2958.2007.05898.x 17880423

[B26] Zhang B Shih JW Wear DJ Tsai S Lo SC High-level expression of H-ras and c-myc oncogenese in mycoplasm-mediated malignant cell transformation Proc Soc Exp Biol Med 1997 214 359 66 9111527 10.3181/00379727-214-44104

[B27] Tsai S Wear DJ Shih JWK Lo SC Mycoplasmas and oncogenesis: Persistent infection and multistage malignant transformation. Proc Natl Acad Sci USA 1995 92 10197 201 10.1073/pnas.92.22.10197 7479753 PMC40763

[B28] Hayward SW Dahiya R Cunha GR Bartek J Deshpande N Narayan P Establishment and characterization of an immortalized but non-transformed human prostate epithelial cell line: BPH-1 Vitro Cell Dev Biol Anim 1995 31 14 24 10.1007/BF02631333 7535634

